# The impact of methadone maintenance therapy on access to regular physician care regarding hepatitis C among people who inject drugs

**DOI:** 10.1371/journal.pone.0194162

**Published:** 2018-03-26

**Authors:** Lianping Ti, María Eugenia Socías, Evan Wood, M-J Milloy, Ekaterina Nosova, Kora DeBeck, Thomas Kerr, Kanna Hayashi

**Affiliations:** 1 British Columbia Centre for Excellence in HIV/AIDS, Vancouver, BC, Canada; 2 Department of Medicine, University of British Columbia, Vancouver, BC, Canada; 3 School of Public Policy, Simon Fraser University, Suite, Vancouver, BC Canada; 4 Faculty of Health Sciences, Simon Fraser University, University Drive, Burnaby, BC, Canada; Centers for Disease Control and Prevention, UNITED STATES

## Abstract

**Background & aims:**

People who inject drugs (PWID) living with hepatitis C virus (HCV) infection often experience barriers to accessing HCV treatment and care. New, safer and more effective direct-acting antiviral-based therapies offer an opportunity to scale-up HCV-related services. Methadone maintenance therapy (MMT) programs have been shown to be effective in linking PWID to health and support services, largely in the context of HIV. The objective of the study was to examine the relationship between being enrolled in MMT and having access to regular physician care regarding HCV among HCV antibody-positive PWID in Vancouver, Canada.

**Design:**

Three prospective cohort studies of people who use illicit drugs.

**Setting:**

Vancouver, Canada.

**Participants:**

We restricted the study sample to 1627 HCV-positive PWID between September 2005 and May 2015.

**Measurements:**

A marginal structural model using inverse probability of treatment weights was used to estimate the longitudinal relationship between being enrolled in MMT and having a regular HCV physician and/or specialist.

**Findings:**

In total, 1357 (83.4%) reported having access to regular physician care regarding HCV at least once during the study period. A marginal structural model estimated a 2.12 (95% confidence interval [CI]: 1.77–2.20) greater odds of having a regular HCV physician among participants enrolled in MMT compared to those not enrolled.

**Conclusions:**

HCV-positive PWID who enrolled in MMT were more likely to report access to regular physician care regarding HCV compared to those not enrolled in MMT. These findings demonstrate that opioid agonist treatment may be helpful in linking PWID to HCV care, and highlight the need to better engage people who use drugs in substance use care, when appropriate.

## Introduction

Hepatitis C virus (HCV) is a significant burden among people who inject drugs (PWID) globally, with prevalence estimates of approximately 60–80% [1]. Of concern, previous studies have shown that PWID are at greater risk of HCV infection and transmission due to a combination of individual factors and social-structural exposures, including high prevalence of poly-substance use, comorbidities, poor access to health care, socioeconomic marginalization, and the persistence of enforcement-based rather than health-based responses to illicit drug use [1–4]. Moreover, chronic HCV infection has become one of the largest contributors to the loss of health-adjusted life years, liver-related morbidity, and mortality among this population [5,6].

Linkage to treatment and care are important considerations for addressing the HCV epidemic, particularly as countries move towards universal coverage of direct-acting antiviral (DAA)-based therapy to treat HCV infection [7–9]. During the interferon-based treatment era, however, studies conducted in developed countries have shown that only 1–6% of this population have ever been treated, with fewer having been linked to care [10–12]. Given that many individuals living with HCV have underlying comorbid conditions and other social-structural issues that may complicate care delivery, interventions that seek to improve the HCV care continuum should take into account a comprehensive and multidisciplinary approach to treatment and care [13,14].

Methadone maintenance therapy (MMT) has been consistently shown to promote uptake of other medical treatment and care, particularly in the context of HIV infection. For example, studies have demonstrated an increase in uptake of antiretroviral therapy (ART) among PWID who are on MMT [15,16]. Other research has demonstrated the beneficial impact of MMT in retaining people on ART, resulting in high rates of adherence and viral load suppression [17]. Positive HCV-related outcomes have also been observed, with previous studies suggesting that opioid agonist therapy (OAT) was negatively associated with time to HCV reinfection [18]. However, little is known about the impact of MMT on other HCV-related outcomes, particularly as it relates to linkage to care among this population [19]. Therefore, the objective of this study was to examine the relationship between MMT and having access to regular physician care regarding HCV among HCV-positive PWID.

## Methods

### Study design

The Vancouver Injection Drug Users Study (VIDUS), AIDS Care Cohort to evaluate Exposure to Survival Services (ACCESS), and the At-Risk Youth Study (ARYS), are three open prospective cohort studies of people who use illicit drugs in Vancouver, Canada. The participants have been recruited through self-referral, word-of-mouth, and street outreach. These cohorts, including their specific eligibility criteria, have been described in detail previously [20–22]. In brief, VIDUS consists of HIV-negative adult (≥18 years of age) PWID. All participants must have injected an illicit drug in the previous month to be eligible for inclusion. ACCESS is a cohort of HIV-positive adults people who use illicit drugs (PWUD) who must have recently used an illicit drug other than or in addition to cannabis in the month prior to enrollment. Similarly, ARYS consists of street-involved youth between the ages of 14 and 26 who have used illicit drugs other than or in addition to cannabis in the month prior to enrollment. In addition, all individuals must have resided in the greater Vancouver region and provided written informed consent to be eligible for the study.

At baseline and semi-annually, participants completed a harmonized interviewer-administered questionnaire that elicited information on socio-demographic characteristics, drug use patterns, involvement in addiction treatment, and other relevant exposures and outcomes (i.e., participants in the VIDUS, ACCESS, and ARYS studies completed an identical questionnaire to allow for pooled analyses and comparisons across cohorts). Additionally, at each study visit, participants provided blood samples for HIV and HCV serologic tests and HIV disease monitoring as appropriate. Participants were compensated for each study visit ($30 CDN). The VIDUS, ACCESS, and ARYS studies were approved by the University of British Columbia/Providence Health Care Research Ethics Board.

### Study sample

The study was conducted between September 2005 and May 2015 and the sample was restricted to participants who: 1) were HCV seropositive at baseline or became positive during follow-up via serologic test; 2) completed at least one follow-up visit after the HCV-positive test result; 3) reported a history of injection drug use at a visit when their blood sample tested positive for HCV; 4) did not die during the study period (or up until the most recent date of death confirmed through a confidential linkage to the provincial Vital Statistics database); 5) has chronic HCV, defined via self-report as those who reported not having naturally cleared HCV, which was derived through a series of questions related to HCV treatment completion and testing results.

### Variable selection

The primary outcome of interest was having access to regular physician care regarding HCV, defined as responding ‘yes’ to either of the following questions: “Do you have a doctor that you see regularly about your Hep C (at least once in the last six months)?” or “Have you seen a specialist doctor about your Hep C in the last six months?” We chose to examine HCV care more broadly and did not restrict to treatment access given that there are currently fibrosis level restrictions to prescribing DAA medications; nonetheless, we felt it was important that HCV care include both access to a general and specialist doctor given that in the BC context, treatment guidelines indicate that both primary care practitioners with experience providing treatment and specialists are able to prescribe DAA treatment to patients. Moreover, we recognize that even if individuals are not able to access treatment due to these restrictions, linkage to care remains an important outcome measure. The question specifically asks whether participants have a doctor that they see regularly about their HCV and therefore, it is assumed that the patient-provider interaction occurred more than once. The main explanatory variable of interest was having been enrolled in MMT in the last six months.

We also considered a selection of other confounders hypothesized to be associated with the main independent and outcome variables, including: sex (male vs. female); age (per year increase); HIV serostatus (positive vs. negative); homelessness (yes vs. no); daily opioid injection, including heroin or prescription opioid injection (≥ daily vs. < daily); daily stimulant injection, including cocaine, crack cocaine, or crystal methamphetamine injection (≥ daily vs. < daily); stable employment, defined as having a regular job, temporary work, or self-employed (yes vs. no); hospital use (yes vs. no); and incarceration (yes vs. no). All variables except for sex were time-updated and referred to the six-month period prior to the follow-up interview unless otherwise indicated.

### Statistical analyses

First, we compared the characteristics of PWID who reported having access to regular physician care regarding HCV at least once during the study period to those who did not; Pearson’s Chi-square test (for categorical variables) and the Mann-Whitney U test (for continuous variables) were used as significance tests for the statistics calculated. Then, we constructed a marginal structural model with inverse probability of treatment weights (IPTW), which can handle time-dependent variables that are simultaneously confounders of the effect of interest and are also predicted by previous treatment (i.e., being on MMT), and can also adjust for selection bias [23]. Specifically, this approach can model the relationship between the explanatory and outcome variables by correcting for the non-random assignment of the treatment. Prior to calculating the weights, all time-dependent confounders were lagged to ensure that the confounders occurred before being enrolled in MMT. We then computed the stabilized IPTW using pooled logistic regression. We chose to use stabilized weights given that unstabilized weights can potentially lead to estimators with large variance [23,24]. We considered the confounding variables listed above for calculating weights, and used bootstrapping methods based on 100 bootstrapped runs to create confidence intervals (CI) for the estimates. A regression model was used the estimate the effect of being enrolled in MMT on having access to regular physician care regarding HCV after adjusting for the stabilized weights calculated.

As a sensitivity analysis, we constructed unadjusted and adjusted unweighted estimates of the effect of being enrolled in MMT on having access to regular physician care regarding HCV using mixed effects logistic regression (MELR) modelling. Specifically, we lagged all confounding variables and included them in the multivariable model regardless of significance in bivariable analyses given that they were *a-priori* variables known to be confounders in the relationship between being enrolled in MMT and having access to regular physician care regarding HCV. CIs were calculated based on Wald statistics. All statistical analyses were executed using R version 0.99.892 (R Foundation for Statistical Computing, Vienna, Austria). All p-values were two-sided.

## Results

Between September 2005 and May 2015, 1627 HCV-positive PWID met the inclusion criteria for the analyses; there were 573 (35.2%) females and the median age at baseline was 41 years (quartile [Q]1 –Q3: 34–47 years). The majority of participants in the study had a history of opioid use or had a history of being on opioid agonist treatment (95.2%). Over the study period, participants contributed 9212 person-years of follow-up, and the median length of follow-up was 6.83 years (Q1 –Q3: 2.71–8.62 years). In total, 1357 (83.4%) participants reported having access to regular physician care regarding HCV at least once during the study period; more specifically, 1336 (82.1%) reported seeing a doctor regularly about their HCV and 637 (39.2%) reported seeing a specialist about their HCV at least once during the study period (not mutually exclusive). Table 1 presents baseline characteristics of study participants, stratified by having access to regular HCV physician care at least once during the study period.

**Table 1:** Baseline characteristics of HCV-positive people who inject drugs in Vancouver, stratified by having regular physician care regarding HCV at least once during study period.

Characteristic	Total (%) (*n* = 1627)	Access to regular physician care regarding HCV		*p—*value
		Yes (*%*) (*n* = 1357)	No (%) (*n* = 270)	
Enrollment in MMT*	666 (40.9)	597 (44.0)	69 (25.6)	<0.001
Male	1054 (64.8)	881 (64.9)	173 (64.1)	0.790
Age (med, Q1-Q3)	41 (34–47)	42 (35–48)	34 (26–42)	<0.001
HIV-positive serostatus	580 (35.6)	523 (38.5)	57 (21.1)	<0.001
Stable employment*	370 (22.7)	309 (22.8)	61 (22.6)	0.949
Homelessness*	626 (38.5)	477 (35.2)	149 (55.2)	<0.001
Daily opioid injection*	530 (32.6)	430 (31.7)	100 (37.0)	0.084
Daily stimulant injection*	238 (14.6)	190 (14.0)	48 (17.8)	0.115
Hospital use*	315 (19.4)	267 (19.7)	48 (17.8)	0.471
Incarceration*	298 (18.3)	232 (17.1)	66 (24.4)	0.006

HCV: hepatitis C virus; MMT: methadone maintenance therapy; Q: quartile

* Refers to the six-month period prior to the interview

Table 2 presents the results of the unweighted and weighted estimates of the effect of being enrolled in MMT on having access to regular physician care regarding HCV. The marginal structural model using IPTW to adjust for time-updated weights yielded an adjusted odds ratio (AOR) of 2.12 (95%CI: 1.77–2.20). In sensitivity analyses, using MELR methods, the unadjusted odds of having access to regular physician care regarding HCV among participants enrolled in MMT was 2.18 times higher (95%CI: 1.95–2.44) compared to those who were not on MMT. Adjusting for various demographic, behavioural, and social-structural exposures, a multivariable MELR model indicated a 2.11 higher odds of having access to regular physician care regarding HCV among participants enrolled in MMT (95%CI: 1.89–2.36) compared to those who were not on MMT.

**Table 2:** Regression analyses on the effect of being enrolled in methadone maintenance therapy on having access to regular physician care regarding HCV among HCV-positive people who inject drugs (n = 1627).

Model specification	Measure of effect [OR (95% CI)]
*Unweighted estimates*	
Unadjusted, generalized linear mixed effect model	2.18 (1.95–2.44)
Adjusted, generalized linear mixed effect model*	2.11 (1.89–2.36)
*Weighted estimates*	
Marginal structural model with IPTW	2.12 (1.77–2.20)

OR: odds ratio; CI: confidence interval; IPTW: inverse probability of treatment weights

*Model adjusted for age, gender, HIV, homelessness, daily opioid injection, daily stimulant injection, employment, and jail. Confounder variables were lagged to assess temporality.

## Discussion

In the present study, we found that a substantial proportion of a community-recruited sample of HCV-positive PWID reported having access to regular physician care regarding HCV. Furthermore, we found an independent association between being on MMT and having access to regular physician care regarding HCV, even after adjusting for a range of confounders. The findings from this study are consistent with previous research focused on HIV care that highlighted the positive role that MMT plays in ART retention, adherence, and viral load suppression among HIV-positive PWID [17,25,26]. Specifically, a randomized controlled trial found that PWID enrolled in a MMT program reported significantly faster entry into HIV care compared to those without a history of MMT (hazard ratio = 2.97; 95%CI: 1.20–6.21) [27]. Our findings add to the literature by suggesting that the benefits of opioid substitution treatment programs, such as MMT, may also be extended to the HCV context given the associated effectiveness in linking PWID to HCV care.

There are a number of possible pathways that underline the relationship between engagement in MMT and accessing HCV care. First, it is likely that access to addiction treatment gives physicians an opportunity to discuss HCV testing, treatment, and care options and can provide an entry point for the delivery of these services to patients [28,29]. Second, MMT is generally daily dispensed through pharmacies in BC; thus, community pharmacists are uniquely positioned to link PWID to HCV testing and treatment [30]. Given that little is known regarding other possible pathways, future research is needed to more clearly understand the relationship between engagement in MMT and accessing HCV care.

There is a growing body of evidence that suggests that the integration of infectious disease testing, treatment, and care within addiction treatment programs is associated with better health outcomes and increased utilization of healthcare services among PWID and other marginalized populations [13,14]. Indeed, the co-location of addiction treatment, harm reduction, and infectious disease services have been successfully applied to settings with a high prevalence of HCV, including in correctional and substance use treatment facilities [13,29,31]. As such, a number of international guidelines focused on HCV management and care have recommended integrated, multidisciplinary approaches to scale up testing and treatment services [32,33]. Given the increasing availability and coverage of DAA therapy in BC and elsewhere [7–9], efforts towards identifying models of care that integrate HCV treatment with addiction treatment and other harm reduction strategies in order to reduce HCV- associated morbidity and mortality, and secondarily, prevent transmission, are needed.

The present study has several limitations. First, the observational nature of the study design limited our ability to determine a direct causal relationship between being enrolled in MMT and having access to regular physician care regarding HCV. However, we attempted to address this through analytical methods by constructing a marginal structural model to adjust for time-dependent confounding to estimate the causal effect of being enrolled in MMT on the outcome. Marginal structural modelling is often used for causal inference in observational studies to allow for improved adjustment of confounding in situations where time-dependent confounders are also affected by previous exposure. To address the issue of temporality, we also lagged the explanatory variables to ensure that these were captured prior to the outcome. Second, the study included some data derived from self-report and thus, may be subject to reporting biases. Specifically, participants may have responded as having access to regular physician care regarding HCV if their addiction medicine doctor who prescribed their methadone asked them casually about their HCV once during a six-month period. This may have biased our results away from the null; however, as there is movement towards multidisciplinary care for PWID, we feel that this information would still be important to capture. Third, there may be unmeasured confounding given that we were only able to control for known confounders. Fourth, we measured chronic HCV status based on HCV-antibody positive test results and self-reports of having never been told by a physician that they no longer have HCV but were not on HCV treatment. Therefore, it is unknown whether these individuals had active HCV infection during the study period. Lastly, our study was not randomly recruited and therefore may not be representative of local PWID or generalizable to other PWID populations outside of Vancouver.

In sum, the present study found that HCV-positive PWID who enrolled in MMT were more likely to have accessed regular physician care regarding HCV compared to those not enrolled in MMT. Our findings demonstrate that MMT programs may be effective for linking PWID to HCV care, and highlight the need to engage PWID in addiction treatment, when appropriate.
